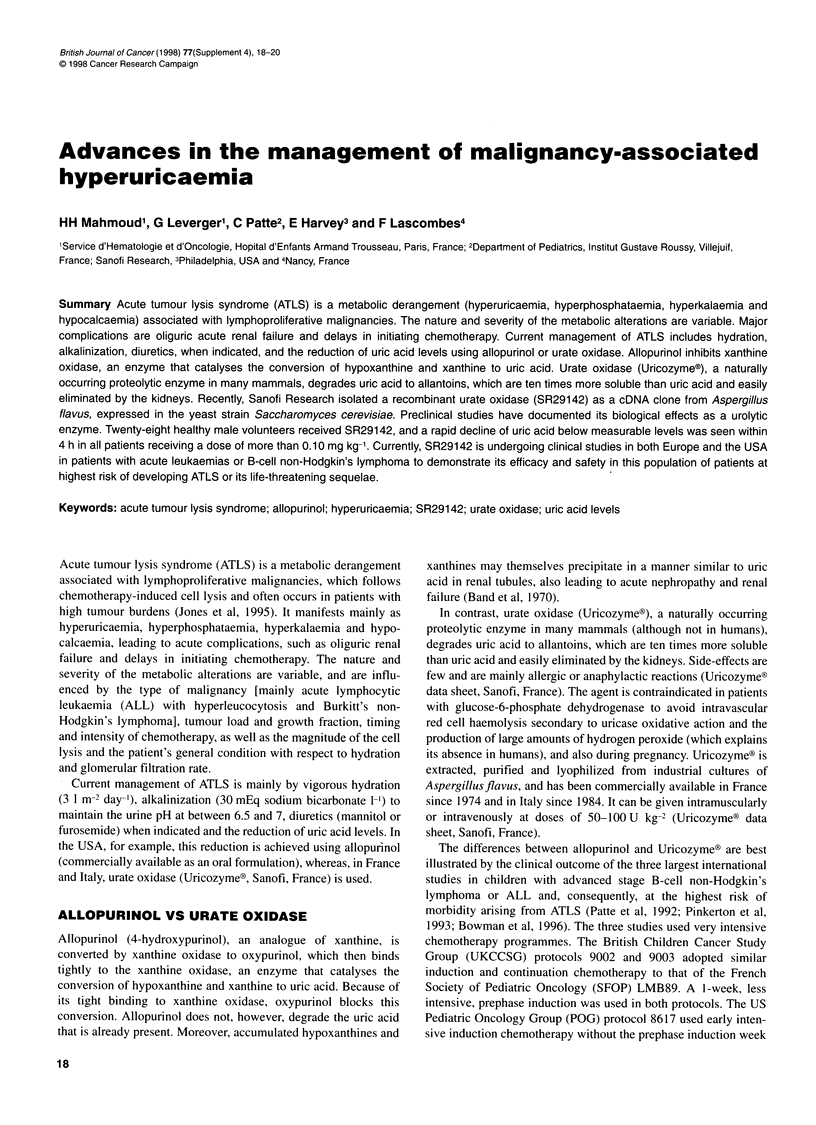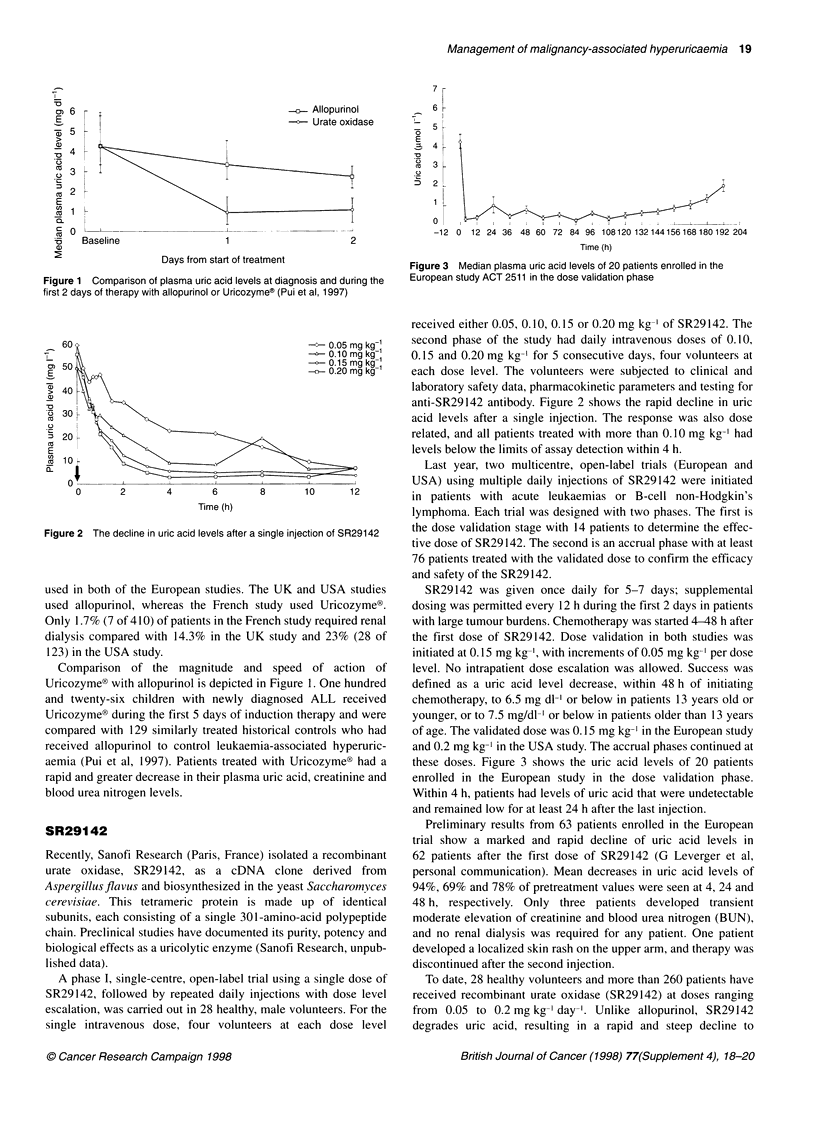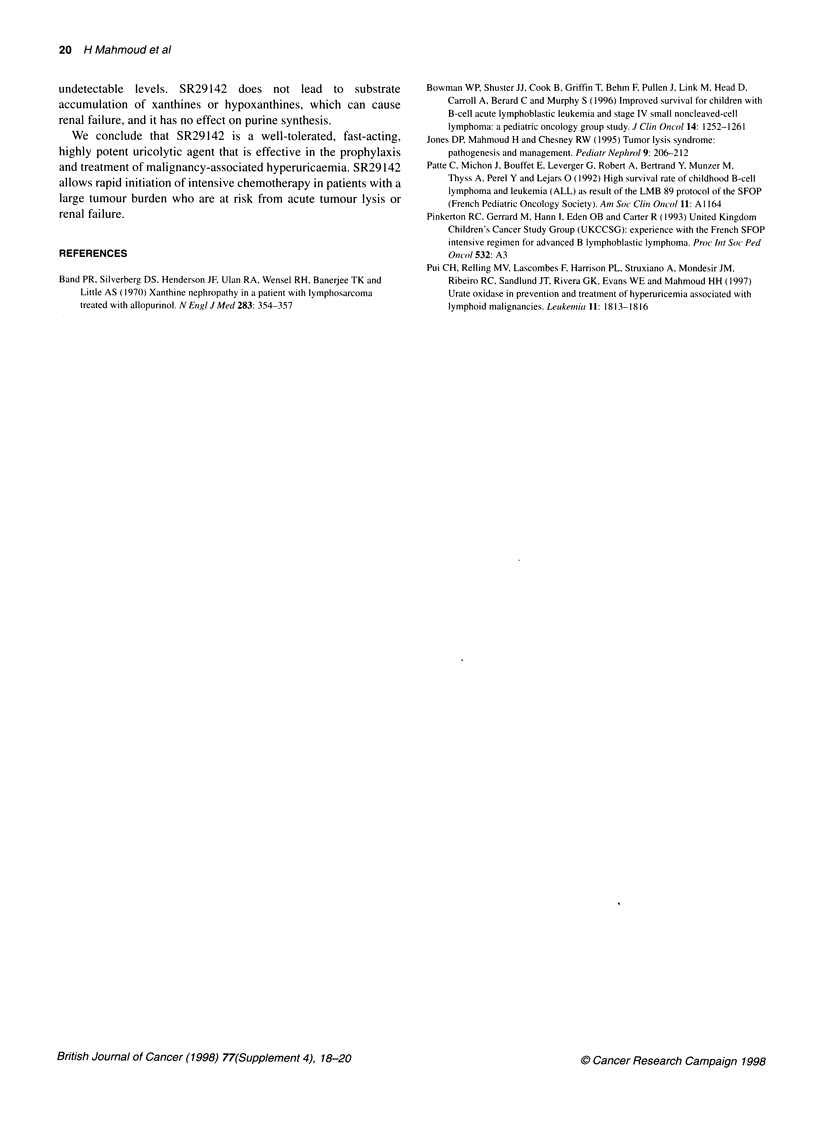# Advances in the management of malignancy-associated hyperuricaemia.

**DOI:** 10.1038/bjc.1998.432

**Published:** 1998-06

**Authors:** H. H. Mahmoud, G. Leverger, C. Patte, E. Harvey, F. Lascombes

**Affiliations:** Service d'Hematologie et d'Oncologie, Hopital d'Enfants Armand Trousseau, Paris, France.

## Abstract

Acute tumour lysis syndrome (ATLS) is a metabolic derangement (hyperuricaemia, hyperphosphataemia, hyperkalaemia and hypocalcaemia) associated with lymphoproliferative malignancies. The nature and severity of the metabolic alterations are variable. Major complications are oliguric acute renal failure and delays in initiating chemotherapy. Current management of ATLS includes hydration, alkalinization, diuretics, when indicated, and the reduction of uric acid levels using allopurinol or urate oxidase. Allopurinol inhibits xanthine oxidase, an enzyme that catalyses the conversion of hypoxanthine and xanthine to uric acid. Urate oxidase (Uricozyme), a naturally occurring proteolytic enzyme in many mammals, degrades uric acid to allantoins, which are ten times more soluble than uric acid and easily eliminated by the kidneys. Recently, Sanofi Research isolated a recombinant urate oxidase (SR29142) as a cDNA clone from Aspergillus flavus, expressed in the yeast strain Saccharomyces cerevisiae. Preclinical studies have documented its biological effects as a urolytic enzyme. Twenty-eight healthy male volunteers received SR29142, and a rapid decline of uric acid below measurable levels was seen within 4 h in all patients receiving a dose of more than 0.10 mg kg(-1). Currently, SR29142 is undergoing clinical studies in both Europe and the USA in patients with acute leukaemias or B-cell non-Hodgkin's lymphoma to demonstrate its efficacy and safety in this population of patients at highest risk of developing ATLS or its life-threatening sequelae.


British Journal of Cancer (1998) 77(Supplement 4), 18–20
© 1998 Cancer Research Campaign

### Advances in the management of malignancy-associated hyperuricaemia

**HH Mahmoud[1], G Leverger[1], C Patte[2], E Harvey[3] and F Lascombes[4]**

[1]Service d'Hematologie et d'Oncologie, Hopital d'Enfants Armand Trousseau, Paris, France; [2]Department of Pediatrics, Institut Gustave Roussy, Villejuif, France; Sanofi Research, [3]Philadelphia, USA and [4]Nancy, France

**Summary** Acute tumour lysis syndrome (ATLS) is a metabolic derangement (hyperuricaemia, hyperphosphataemia, hyperkalaemia and hypocalcaemia) associated with lymphoproliferative malignancies. The nature and severity of the metabolic alterations are variable. Major complications are oliguric acute renal failure and delays in initiating chemotherapy. Current management of ATLS includes hydration, alkalinization, diuretics, when indicated, and the reduction of uric acid levels using allopurinol or urate oxidase. Allopurinol inhibits xanthine oxidase, an enzyme that catalyses the conversion of hypoxanthine and xanthine to uric acid. Urate oxidase (Uricozyme®), a naturally occurring proteolytic enzyme in many mammals, degrades uric acid to allantoins, which are ten times more soluble than uric acid and easily eliminated by the kidneys. Recently, Sanofi Research isolated a recombinant urate oxidase (SR29142) as a cDNA clone from *Aspergillus flavus*, expressed in the yeast strain *Saccharomyces cerevisiae*. Preclinical studies have documented its biological effects as a urolytic enzyme. Twenty-eight healthy male volunteers received SR29142, and a rapid decline of uric acid below measurable levels was seen within 4 h in all patients receiving a dose of more than 0.10 mg $kg^{-1}$. Currently, SR29142 is undergoing clinical studies in both Europe and the USA in patients with acute leukaemias or B-cell non-Hodgkin's lymphoma to demonstrate its efficacy and safety in this population of patients at highest risk of developing ATLS or its life-threatening sequelae.

**Keywords:** acute tumour lysis syndrome; allopurinol; hyperuricaemia; SR29142; urate oxidase; uric acid levels

Acute tumour lysis syndrome (ATLS) is a metabolic derangement associated with lymphoproliferative malignancies, which follows chemotherapy-induced cell lysis and often occurs in patients with high tumour burdens (Jones et al, 1995). It manifests mainly as hyperuricaemia, hyperphosphataemia, hyperkalaemia and hypocalcaemia, leading to acute complications, such as oliguric renal failure and delays in initiating chemotherapy. The nature and severity of the metabolic alterations are variable, and are influenced by the type of malignancy [mainly acute lymphocytic leukaemia (ALL) with hyperleucocytosis and Burkitt's non-Hodgkin's lymphoma], tumour load and growth fraction, timing and intensity of chemotherapy, as well as the magnitude of the cell lysis and the patient's general condition with respect to hydration and glomerular filtration rate.

Current management of ATLS is mainly by vigorous hydration (3 l $m^{-2}$ $day^{-1}$), alkalinization (30 mEq sodium bicarbonate $l^{-1}$) to maintain the urine pH at between 6.5 and 7, diuretics (mannitol or furosemide) when indicated and the reduction of uric acid levels. In the USA, for example, this reduction is achieved using allopurinol (commercially available as an oral formulation), whereas, in France and Italy, urate oxidase (Uricozyme®, Sanofi, France) is used.

### ALLOPURINOL VS URATE OXIDASE

Allopurinol (4-hydroxypurinol), an analogue of xanthine, is converted by xanthine oxidase to oxypurinol, which then binds tightly to the xanthine oxidase, an enzyme that catalyses the conversion of hypoxanthine and xanthine to uric acid. Because of its tight binding to xanthine oxidase, oxypurinol blocks this conversion. Allopurinol does not, however, degrade the uric acid that is already present. Moreover, accumulated hypoxanthines and xanthines may themselves precipitate in a manner similar to uric acid in renal tubules, also leading to acute nephropathy and renal failure (Band et al, 1970).

In contrast, urate oxidase (Uricozyme®), a naturally occurring proteolytic enzyme in many mammals (although not in humans), degrades uric acid to allantoins, which are ten times more soluble than uric acid and easily eliminated by the kidneys. Side-effects are few and are mainly allergic or anaphylactic reactions (Uricozyme® data sheet, Sanofi, France). The agent is contraindicated in patients with glucose-6-phosphate dehydrogenase to avoid intravascular red cell haemolysis secondary to uricase oxidative action and the production of large amounts of hydrogen peroxide (which explains its absence in humans), and also during pregnancy. Uricozyme® is extracted, purified and lyophilized from industrial cultures of *Aspergillus flavus*, and has been commercially available in France since 1974 and in Italy since 1984. It can be given intramuscularly or intravenously at doses of 50–100 U $kg^{-2}$ (Uricozyme® data sheet, Sanofi, France).

The differences between allopurinol and Uricozyme® are best illustrated by the clinical outcome of the three largest international studies in children with advanced stage B-cell non-Hodgkin's lymphoma or ALL and, consequently, at the highest risk of morbidity arising from ATLS (Patte et al, 1992; Pinkerton et al, 1993; Bowman et al, 1996). The three studies used very intensive chemotherapy programmes. The British Children Cancer Study Group (UKCCSG) protocols 9002 and 9003 adopted similar induction and continuation chemotherapy to that of the French Society of Pediatric Oncology (SFOP) LMB89. A 1-week, less intensive, prephase induction was used in both protocols. The US Pediatric Oncology Group (POG) protocol 8617 used early intensive induction chemotherapy without the prephase induction week

**Figure 1** Comparison of plasma uric acid levels at diagnosis and during the first 2 days of therapy with allopurinol or Uricozyme® (Pui et al, 1997)

**Figure 3** Median plasma uric acid levels of 20 patients enrolled in the European study ACT 2511 in the dose validation phase

**Figure 2** The decline in uric acid levels after a single injection of SR29142

used in both of the European studies. The UK and USA studies used allopurinol, whereas the French study used Uricozyme®. Only 1.7% (7 of 410) of patients in the French study required renal dialysis compared with 14.3% in the UK study and 23% (28 of 123) in the USA study.

Comparison of the magnitude and speed of action of Uricozyme® with allopurinol is depicted in Figure 1. One hundred and twenty-six children with newly diagnosed ALL received Uricozyme® during the first 5 days of induction therapy and were compared with 129 similarly treated historical controls who had received allopurinol to control leukaemia-associated hyperuricaemia (Pui et al, 1997). Patients treated with Uricozyme® had a rapid and greater decrease in their plasma uric acid, creatinine and blood urea nitrogen levels.

### SR29142

Recently, Sanofi Research (Paris, France) isolated a recombinant urate oxidase, SR29142, as a cDNA clone derived from *Aspergillus flavus* and biosynthesized in the yeast *Saccharomyces cerevisiae*. This tetrameric protein is made up of identical subunits, each consisting of a single 301-amino-acid polypeptide chain. Preclinical studies have documented its purity, potency and biological effects as a uricolytic enzyme (Sanofi Research, unpublished data).

A phase I, single-centre, open-label trial using a single dose of SR29142, followed by repeated daily injections with dose level escalation, was carried out in 28 healthy, male volunteers. For the single intravenous dose, four volunteers at each dose level received either 0.05, 0.10, 0.15 or 0.20 mg kg$^{-1}$ of SR29142. The second phase of the study had daily intravenous doses of 0.10, 0.15 and 0.20 mg kg$^{-1}$ for 5 consecutive days, four volunteers at each dose level. The volunteers were subjected to clinical and laboratory safety data, pharmacokinetic parameters and testing for anti-SR29142 antibody. Figure 2 shows the rapid decline in uric acid levels after a single injection. The response was also dose related, and all patients treated with more than 0.10 mg kg$^{-1}$ had levels below the limits of assay detection within 4 h.

Last year, two multicentre, open-label trials (European and USA) using multiple daily injections of SR29142 were initiated in patients with acute leukaemias or B-cell non-Hodgkin's lymphoma. Each trial was designed with two phases. The first is the dose validation stage with 14 patients to determine the effective dose of SR29142. The second is an accrual phase with at least 76 patients treated with the validated dose to confirm the efficacy and safety of the SR29142.

SR29142 was given once daily for 5–7 days; supplemental dosing was permitted every 12 h during the first 2 days in patients with large tumour burdens. Chemotherapy was started 4–48 h after the first dose of SR29142. Dose validation in both studies was initiated at 0.15 mg kg$^{-1}$, with increments of 0.05 mg kg$^{-1}$ per dose level. No intrapatient dose escalation was allowed. Success was defined as a uric acid level decrease, within 48 h of initiating chemotherapy, to 6.5 mg dl$^{-1}$ or below in patients 13 years old or younger, or to 7.5 mg/dl$^{-1}$ or below in patients older than 13 years of age. The validated dose was 0.15 mg kg$^{-1}$ in the European study and 0.2 mg kg$^{-1}$ in the USA study. The accrual phases continued at these doses. Figure 3 shows the uric acid levels of 20 patients enrolled in the European study in the dose validation phase. Within 4 h, patients had levels of uric acid that were undetectable and remained low for at least 24 h after the last injection.

Preliminary results from 63 patients enrolled in the European trial show a marked and rapid decline of uric acid levels in 62 patients after the first dose of SR29142 (G Leverger et al, personal communication). Mean decreases in uric acid levels of 94%, 69% and 78% of pretreatment values were seen at 4, 24 and 48 h, respectively. Only three patients developed transient moderate elevation of creatinine and blood urea nitrogen (BUN), and no renal dialysis was required for any patient. One patient developed a localized skin rash on the upper arm, and therapy was discontinued after the second injection.

To date, 28 healthy volunteers and more than 260 patients have received recombinant urate oxidase (SR29142) at doses ranging from 0.05 to 0.2 mg kg$^{-1}$ day$^{-1}$. Unlike allopurinol, SR29142 degrades uric acid, resulting in a rapid and steep decline to

© Cancer Research Campaign 1998

undetectable levels. SR29142 does not lead to substrate accumulation of xanthines or hypoxanthines, which can cause renal failure, and it has no effect on purine synthesis.

We conclude that SR29142 is a well-tolerated, fast-acting, highly potent uricolytic agent that is effective in the prophylaxis and treatment of malignancy-associated hyperuricaemia. SR29142 allows rapid initiation of intensive chemotherapy in patients with a large tumour burden who are at risk from acute tumour lysis or renal failure.

### REFERENCES

Band PR, Silverberg DS, Henderson JF, Ulan RA, Wensel RH, Banerjee TK and Little AS (1970) Xanthine nephropathy in a patient with lymphosarcoma treated with allopurinol. *N Engl J Med* **283**: 354–357

Bowman WP, Shuster JJ, Cook B, Griffin T, Behm F, Pullen J, Link M, Head D, Carroll A, Berard C and Murphy S (1996) Improved survival for children with B-cell acute lymphoblastic leukemia and stage IV small noncleaved-cell lymphoma: a pediatric oncology group study. *J Clin Oncol* **14**: 1252–1261

Jones DP, Mahmoud H and Chesney RW (1995) Tumor lysis syndrome: pathogenesis and management. *Pediatr Nephrol* **9**: 206–212

Patte C, Michon J, Bouffet E, Leverger G, Robert A, Bertrand Y, Munzer M, Thyss A, Perel Y and Lejars O (1992) High survival rate of childhood B-cell lymphoma and leukemia (ALL) as result of the LMB 89 protocol of the SFOP (French Pediatric Oncology Society). *Am Soc Clin Oncol* **11**: A1164

Pinkerton RC, Gerrard M, Hann I, Eden OB and Carter R (1993) United Kingdom Children's Cancer Study Group (UKCCSG): experience with the French SFOP intensive regimen for advanced B lymphoblastic lymphoma. *Proc Int Soc Ped Oncol* **532**: A3

Pui CH, Relling MV, Lascombes F, Harrison PL, Struxiano A, Mondesir JM, Ribeiro RC, Sandlund JT, Rivera GK, Evans WE and Mahmoud HH (1997) Urate oxidase in prevention and treatment of hyperuricemia associated with lymphoid malignancies. *Leukemia* **11**: 1813–1816

© Cancer Research Campaign 1998